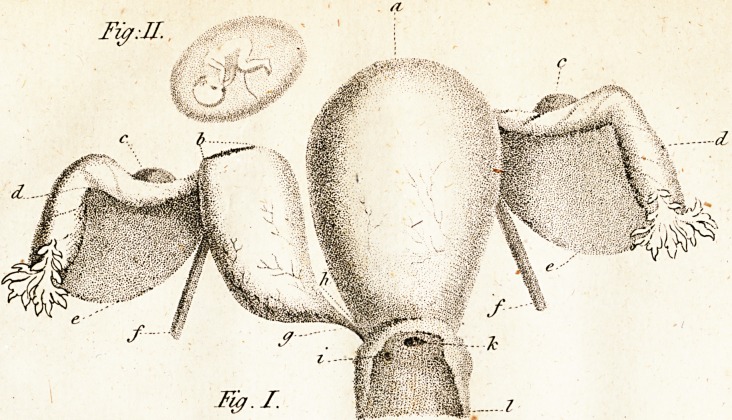# A Case of Double Uterus
*This curious case is extracted from the first volume of a respectable periodical work, entitled "Oberdeutsche Bey" träge zur Naturlehre und Oekonomie, gesammelt und " herausgegeben von *Karl Erenbert von Moll*, Oesterreichi" schern Landmanne, der Gesellschaft Naturforschender " Freunde in Berlin, &c. Mitgliede." 8vo. Salzburg, 1787.—Editor.


**Published:** 1792

**Authors:** Antonio Canestrini

**Affiliations:** Physician to the Imperial Mines at Schwatz in Tyrol.


### XVII. *A Case of double Uterus* *. *By* Antonio Caneſtrini, *Phyſician to the Imperial Mines at Schwatz in Tyrol. Tranſlated from the German.*

MARIA ANNA VOKON, wife of the Overſeer of a Foundry at Fernetz, ſmall of ſtature, but of a healthy conſtitution, was married in the twentieth year of her age, and before the end of a twelvemonth was delivered of a female child, which died two days after its birth. A year and a half afterwards ſhe was delivered of another girl, which ſhe ſuckled, but which lived only five weeks. During each of theſe pregnancies ſhe was in good health, and in both went her full time. The menſes never appeared during pregnancy, but at other times ſhe was ſubject to them rather profuſely, and they generally continued eight days.

* This curious caſe is extracted from the firſt volume of a reſpectable periodical work, entitled "Oberdeutſche Beyträge zur Naturlehre und Oekonomie, geſammelt und herausgegeben von *Karl Erenbert von Moll*, Oeſterreichiſchern Landmanne, der Geſellſchaft Naturforſchender Freunde in Berlin, &c. Mitgliede." 8vo. Salzburg, 1787.—EDITOR.

Two

Two months after the birth of her fecond child, being then in her twenty-fourth year, fhe again became pregnant. In the fourth month of this laft pregnancy fhe fell down as fhe was carrying fome wood, and immediately felt much pain of her left thigh, but which foon fubfided. A few days afterwards, however, when, according to her reckoning, fhe had completed the fourth month of her pregnancy, fhe was fuddenly feized with pains of the belly, which made her cry out violently. This happened on the 19th of May, 1781. Her hufband immediately carried her to bed, and called in one of his female neighbours, who rubbed the abdomen with oil, but this did not at all mitigate the pains, which refembled labour pains, though without being accompanied with any difcharge from the vagina. In the evening of that day fhe had a ftool, after having been three days without fuch an evacuation. The pains, however, ftill continued; and fhe was twice feized with vomiting. At fix o'clock the next morning fhe appeared to be in a dying ftate, and about feven fhe expired.

The relations of the deceafed, ftruck with the fudden and fatal termination of the cafe, and obferving that the pains had been confined to the abdomen ; that ſhe had twice been ſeized with vomiting, and that the abdomen ſwelled after her death, were ſuſpicious that her death might have been occaſioned by poiſon. They, therefore, reſolved to have the body opened, and I was called upon for this purpoſe.

After dividing the integuments from the ſternum to the pubis, and opening the cavity of the belly, I found therein a conſiderable quantity of extravaſated blood, of which about two pounds were removed before it was poſſible for me to examine the ſtate of the viſcera.

The omentum was of the uſual ſize, and moderately fat ; the ſtomach exhibited no marks of inflammation, and the liver, gall bladder, and inteſtines, appeared to be in a ſound ſtate. In order to examine theſe parts properly, it became neceſſary to remove about three pounds more of extravaſated blood, and in doing this my hand met with a round and very elaſtic body, which was carefully extracted, and proved to be an ovum with its membranes entire. I next diſcovered that the uterus was lacerated on the right ſide, at its fundus, to the extent of an inch and a half ; and in order to examine this part more accurately, I made an inciſion into it, from from the place where it was torn to its neck, (fee the reference at *g*. Fig. 1. Plate II.)

Upon laying open the cavity of the uterus I found the placenta ftill fo firmly adhering to its inner furface, that I was unable to detach it without tearing it. The uterus was three inches and a half long; its breadth, at its upper part, was two inches and a half, and at its lower part one inch and a half; near its neck it was of the thicknefs of a little finger; but at its fundus, where it was torn, it was not more than a fourth part fo thick. Externally it was only flightly red, but internally it was much more fo, and covered with innumerable fmall veffels and fibres; but it no where exhibited any appearance of gangrene. The laceration extended exactly acrofs the fundus uteri.

It had only one Fallopian tube, and that was on the right fide, and contiguous to the laceration. Through its outer extremity I was able to introduce a probe, which paffed into the cavity of the uterus. There was likewife only one ovarium, but it was larger than ufual, and weighed two drachms and twenty-two grains. The broad and round ligaments, which likewife were to be found only on this fide, were of their natural fize and figure. On the left fide I

 could could difcover no appearance of ovarium, Fallopian tube, or ligaments. This uterus did not feem to be connected with the vagina, nor was there any appearance of an os uteri, but it terminated in a fort of round ligament, or neck, which in length was about a finger's breadth, but in circumference only of about the fize of a little finger. At the upper end of this neck, where I had cut through it, I difcovered two fmall orifices, through which I could with difficulty introduce the probe I had before ufed for the Fallopian tube; but this paffed too little a way to enable me to trace thefe channels to their termination. In other refpects this neck appeared like the reft of the uterus. A confiderable number of blood veffels paffed through it to the body of the uterus, and in cutting through it I divided two of the fize of a common writing pen.

This fingularity of ftructure could not fail to excite my aftonifhment, and a queftion naturally arofe in my mind, How had this woman twice been delivered, when the ftate of the parts feemed to render delivery impoffible? — That I might leave nothing unexamined, I carefully removed from the pelvis all that remained of extravafated blood, and fought for the the place where I had cut through the uterus; and in ſo doing I found that I had only ſeparated it from another uterus, which I now took out, together with part of the vagina connected with it, and remarked what follows:

This ſecond uterus was ſix inches in length; at its upper part four and at its under three inches and a half in breadth, and about half an inch in thickneſs. Its appearance was natural, but its blood veſſels were empty, and its orifice was ſo firmly cloſed, that it was with difficulty I could introduce a probe into its cavity. The vagina was likewiſe in a natural ſtate. Here alſo there were only one Fallopian tube and one ovary. The former was connected with the uterus at its fundus, and on the left ſide, and was of the uſual ſize and appearance. The latter was ſomewhat flatter than the ovary of the other uterus, and weighed only one drachm and forty-two grains. The round ligament here was of the ſame thickneſs as that of the firſt, and the broad ligament was perfectly natural. On the right ſide there was neither ovarium, tube, nor ligaments.

Upon cutting open this uterus there flowed out between two and three ounces of a viſcid, reddiſh fluid; and its inner ſurface exhibited the appearance of numerous and large blood veſſels, but they were empty. In future, to avoid miſtake, I ſhall call this the *larger* uterus, to diſtinguiſh it from the other in which the fœtus lay, and which, from its ſize, I ſhall ſtyle the *ſmaller* uterus.

On the right ſide of the neck of the larger uterus, an inch above the os uteri, was the place where the ſmaller uterus adhered to it, and which I had overlooked, and cut through. I now ſought for the origin of the two channels, the orifices of which I had obſerved in the neck of the ſmaller uterus, and which I now conjectured might paſs through this neck to the larger uterus. The clearing up this matter took me up ſeveral hours. I attempted to paſs firſt a probe, and afterwards merely a hog's briſtle, through this neck into the cavity of the larger uterus; but failing in theſe attempts, I cut through the middle of the larger uterus, in order the better to diſcover the communication. When I had done this, upon carefully examining the place on the inner ſurface of the cavity, immediately oppoſite the part where the outer openings were to be ſeen, I diſcovered, about an inch above the os uteri, a ſmall opening, through which I was able to introduce a

 hog's hog's briſtle. This paſſed from the cavity of the larger uterus to the neck of the ſmaller one; but ſtill the courſe of the other channel remained to be aſcertained, and this proved a ſubject of greater difficulty; for even quickſilver, with which I filled the larger uterus after carefully ſecuring the os uteri by ligature, failed to point it out, the quickſilver making its way out either through the little opening juſt now mentioned, or through the Fallopian tube. At length I examined carefully the outer ſurface of the orifice of the larger uterus, the form of which was perfectly natural; a portion of the vagina was ſtill connected with it, and as I was drawing this and one of the lips of the os uteri from each other, I diſcovered, at the part where the vagina embraces the latter, between the ſides of the vagina and the os uteri, a ſmall funnel-ſhaped orifice, which opened into the vagina. Its orifice was wide enough to admit the head of a large probe; but the channel ſoon became ſo narrow as to allow only a hog's briſtle to go through it. This paſſed under the outer coat of the larger uterus towards the neck of the ſmaller one, where I had before introduced a briſtle into it.

Fig: II.

Fig. I.

Ravenhill Sculp[t]

The fœtus was a male, and ſeemed to be perfectly formed.

### Explanation of Plate II.

### Fig. 1.

*a.* The larger uterus.

*b.* The laceration of the ſmaller uterus, through which the fœtus eſcaped into the cavity of the abdomen.

*c. c.* The ovaria.

*d. d.* The Fallopian tubes.

*e. e.* The broad ligaments.

*f. f.* The round ligaments.

*g.* The neck or ſtem of the ſmaller uterus cut through by the knife.

*h.* The part at which one of the two ſmall channels that paſſed through the neck of the ſmaller uterus opened into the cavity of the larger one.

*i.* The orifice of the other ſmall channel, by means of which a communication was formed between the ſmaller uterus and the vagina.

*k.* The orifice of the larger uterus.

*l.* The vagina cut open lengthwiſe, anteriorly, ſo as to ſhow its communication with each uterus.

### Fig. II.

The fœtus ſurrounded by its membranes.

## Figures and Tables

**Fig. I. Fig: II. f1:**